# Statistical and Machine Learning Link Selection Methods for Brain Functional Networks: Review and Comparison

**DOI:** 10.3390/brainsci11060735

**Published:** 2021-05-31

**Authors:** Ilinka Ivanoska, Kire Trivodaliev, Slobodan Kalajdziski, Massimiliano Zanin

**Affiliations:** 1 Faculty of Computer Science and Engineering, Ss. Cyril and Methodius University, 1000 Skopje, North Macedonia; kire.trivodaliev@finki.ukim.mk (K.T.); slobodan.kalajdziski@finki.ukim.mk (S.K.); 2 Instituto de Física Interdisciplinar y Sistemas Complejos (CSIC-UIB), Campus Universitat de les Illes Balears, E-07122 Palma de Mallorca, Spain; mzanin@ifisc.uib-csic.es

**Keywords:** brain functional networks, link selection, statistics, machine learning

## Abstract

Network-based representations have introduced a revolution in neuroscience, expanding the understanding of the brain from the activity of individual regions to the interactions between them. This augmented network view comes at the cost of high dimensionality, which hinders both our capacity of deciphering the main mechanisms behind pathologies, and the significance of any statistical and/or machine learning task used in processing this data. A link selection method, allowing to remove irrelevant connections in a given scenario, is an obvious solution that provides improved utilization of these network representations. In this contribution we review a large set of statistical and machine learning link selection methods and evaluate them on real brain functional networks. Results indicate that most methods perform in a qualitatively similar way, with NBS (Network Based Statistics) winning in terms of quantity of retained information, AnovaNet in terms of stability and ExT (Extra Trees) in terms of lower computational cost. While machine learning methods are conceptually more complex than statistical ones, they do not yield a clear advantage. At the same time, the high heterogeneity in the set of links retained by each method suggests that they are offering complementary views to the data. The implications of these results in neuroscience tasks are finally discussed.

## 1. Introduction

Since the beginning of modern neuroscience, one of the main focuses has been describing the differences between groups of subjects, with one of them usually comprising people suffering from a given condition, and the other matched healthy control subjects. The objective is to describe what is significantly different between controls and patients, hence what is potentially causing the condition and, ideally, how can its impact be mitigated. Nevertheless, this also yields another benefit, i.e., validation; if no differences are detected in patients suffering from a condition that is profoundly modifying the cognitive capabilities, as e.g., Alzheimer’s or Parkinson’s diseases, one may infer that the data used in the comparison are not characterising important aspects of brain activity.

In recent years, neuroscience has gone through a second shift in its focus. The historical picture of the brain was one of a collection of regions working in a quite independent way, such that a clear association could be established between regions and cognitive tasks—e.g., Broca’s area and speech, or Wernicke’s area and language comprehension [1]. This was nevertheless at odd with two main evidences: brain activation studies, showing that multiple regions seamlessly contribute to simple tasks; and brain plasticity, the process allowing one function to shift to nearby areas in the brain. A new paradigm emerged, in which different areas can work both together and isolated, resulting in respectively integration and segregation of functions and information. While this has long ago been hypothesised, only the recent introduction of complex network theory has made rigorous mathematical studies possible. Complex networks are mathematical objects composed of nodes, pairwise connected by links [2,3,4]. Nodes and links can then be used to respectively represent brain regions and their interactions, giving birth to structural and functional brain networks [5,6].

In spite of important successes, the use of brain network representations also introduced conceptual and computational problems, one of them being the complexity of performing the aforementioned comparison between two groups of subjects. To illustrate, let us consider the simple case of a localised brain tumour. When analysed through fMRI, or even EEG data, a practitioner would locate a region of lower (or null) activity; following the original framework, he/she would deduce which area is affected, and hence which function. Things nevertheless become more complex with the network framework. On one hand, a single region can be (directly or indirectly) involved in many cognitive tasks. On the other hand, and from a computational perspective, the analysis has a higher dimensionality, as it has to be ascertain not just which area is affected, but instead which connections (involving pairs of areas) are. Combining both ideas, it is possible for a damage in a region *a* to affect, e.g., through plasticity, the connectivity between two other regions *b* and *c*, regions that are prima facie not related to *a*.

When the group comparison is performed through automated tools, as e.g., through machine learning models, network representations lead to another problem, commonly called curse of dimensionality [7]. In short, the number of features (here of links, number that scales quadratically with the number of nodes) can become larger than the number of instances, i.e., of subjects available in the analysis; this, in turn, can lead to an over-fitting of the classification models, and hence to unreliable and not generalisable results. The presence of a large number of potentially irrelevant links can further decrease the accuracy of the learning algorithm, and increase memory and computational requirements. Selecting features in brain network analysis can help detecting significant biomarkers for a particular brain disease.

Such increase in complexity can partly be solved through the use of a selection process, i.e., an initial step devoted to identifying only those connections that are relevant for a given study. This is akin to the task of feature selection in machine learning, according to which the initial set of features describing the instances of the problem is filtered to delete those that are not relevant. The practitioner could then disregard the full network, to only focus on the subset of nodes and connections that seem to be related with the condition under study. This not only results in a conceptually simpler problem: it can also drastically reduce the computational complexity of the same, thus allowing the use of more complex numerical techniques in subsequent steps.

Given this state of affair, one fundamental question remains: given the large number of methods proposed in the Literature to perform such selection, how do they compare to each other when applied to brain networks? This paper answers this question through a large-scale review and evaluation of 19 commonly used selection strategies, based on statistical and machine learning principles. To the best of our knowledge, this is the first instance of such a large-scale comparison of methods for feature selection in brain networks. The evaluation is performed using a well-known and publicly available functional Magnetic Resonance Imaging (fMRI) data set, comprising both schizophrenic patients and matched control subjects; and further confirmed with two additional data sets. Methods are ranked according to the quantity of information they retain, measured through the score achieved in a subsequent classification task; their stability, i.e., how consistent is the set of retained links; and their computational cost. Results indicate that most methods perform in a qualitatively similar and efficient way, in the sense that subsequent classification tasks are not negatively affected. Still, the sets of retained links strongly vary across methods; while the same quantity of information is preserved, each method localises it in a different way, thus offering complementary views to the brain structure. Among the best performing methods, it is worth highlight NBS (Network Based Statistics) for the highest quantity of retained information, AnovaNet for the highest stability and ExT (Extra Trees) for the lowest computational cost.

The remainder of the text is organised as follows. Section 2 presents a review of 19 methods for link selection, based on statistical and machine learning principles. Afterwards, Section 3 details the three data sets used in this study, and how the brain networks have been reconstructed from them; and the classification models that have been used to evaluate the effectiveness of the selection methods. Results are presented in Section 4, organised in terms of performance (Section 4.1), stability (Section 4.2), computational cost (Section 4.3) and generalisability (Section 4.4). Lastly, Section 5 draws some conclusions, discusses the limitations of the present study, and sketches future lines of research.

## 2. Methods for Link Selection in Functional Brain Networks

**Figure 1:**
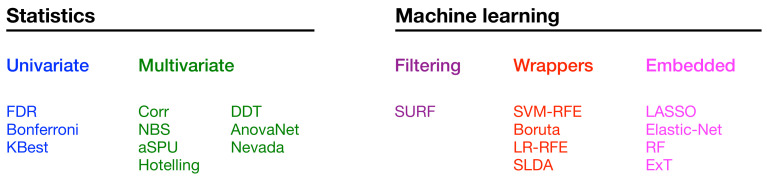
List of the 19 selection methods here considered, classified according to their nature. For the sake of clarity, the same class colour code will be used in all subsequent figures.

As previously introduced, in this work we compare 19 methods—see Figure 1 for a list. These can be divided in two big families, i.e., those based on statistics methods and those based on machine learning models. These two families can further be divided in different groups. Specifically, statistical methods can be univariate, i.e., evaluating one single link at the time (see Section 2.1), or multivariate, when each link is evaluated taking into account the other ones (Section 2.2). On the other hand, machine learning methods can be classified as filtering ones, in which links are selected as an a priori step to classification (Section 2.3.1); wrappers, i.e., when the selection is performed using the result of a classification model (Section 2.3.2); and embedded methods, in which the selection process is performed by looking at the internal process performed by a classification model (Section 2.3.3). Note that this classification is a fuzzy one, and is only meant to provide a general guide on the main underlying principle—to illustrate, any univariate method corrected for multiple comparisons is technically considering all links in the final step, and may therefore be classified as multivariate.

### 2.1. Statistical, Mass Univariate (Local) Approaches

#### 2.1.1. False Discovery Rate (FDR)

The False Discovery Rate (FDR) approach works with uni-dimensional data, such that connections between different pairs of nodes are considered as independent cases. The weights of the corresponding edges of the two samples are compared, for then determining whether the difference is statistically significant for each individual link. This approach uses the adjacent matrix of the brain networks and compares the weights of the links belonging to the lower triangular part of the matrix (matrix half-vectorization). Such comparison can be done through a simple Student’s *t*-test. Here we use the Welch’s *t*-test [8,9], a variation of the *t*-test for cases in which the two samples have different variance.

Testing multiple connections in an independent fashion results in the well-known problem of multiple comparisons. Specifically, the probability of one or more false rejections, also known as type I errors or family-wise error rate (FWER), needs to be controlled. One of the several procedures to address this issue is the False discovery rate (FDR) [10], which provides less stringent type I error control, contributing to greater statistical power. On the other hand, this method also results in an increased number of false rejections (type I errors). This control procedure works by sorting the *p*-values in increasing order, for then finding the largest index *i* satisfying the condition $p_{i}\leq \alpha_{i}/N$ (with i=1,2,…,N, and *N* being the number of tests or edges). In other words, it finds the largest index *i* for which the null hypothesis (the two samples are different) is rejected.

The univariate FDR approach can be summarised in the following steps: (1) calculate the half-vectorization of the brain networks’ adjacency matrices; (2) perform the Weltch’s t-test on each element of the vector; (3) apply the FDR procedure to correct the *p*-values; (4) reject the null hypothesis for each edge if *p*-value < α; (5) sort the edges according to their *p*-value; and (6) extract the significantly different edges.

#### 2.1.2. Bonferroni

As previously discussed, there are several procedures [11,12,13,14,15] that address the issue of multiple comparisons using a correction value, in order to ensure that the FWER is kept below a certain level. One of them is the Bonferroni correction [16], which is a strong control method. This method has a higher probability of producing false negatives, thus reducing the statistical power of the tests. The procedure for link selection using the Bonferroni correction is similar to the previous one, only the FDR is replaced in step 3 by the Bonferroni *p*-values.

#### 2.1.3. Select K Best (KBest)

The KBest method is based on the evaluation of a function (a Chi-squared statistic in our case) that assesses the correlation between the features (i.e., the link weights) and the target variable (the class labels). A fixed and predetermined number *k* of edges with the highest scores are finally selected.

### 2.2. Statistical, Multivariate (Global) Approaches

#### 2.2.1. Correlation of Features (Corr)

This method leverages on the idea that good features are highly correlated with the target variable, but at the same time uncorrelated among themselves. Consequently, if two features are highly correlated, only the one that is more correlated with the target variable is retained. A threshold of 0.5 is used for determining whether pairs of features are linearly correlated between themselves.

#### 2.2.2. Network Based Statistics (NBS)

Network-based statistics (NBS) [17] is a method performing mass-univariate testing on the network connections, with a subsequent control for FWER. The clustering structure of the network, i.e., connected subnetworks in the topological space, is used to correct the initial test statistics and *p*-values, and the result is a set of components that are statistically significant. The test controls the FWER in a weak sense, and gives corrected *p*-values to each subnetwork using permutation testing, in order to conclude whether each component has a statistically significant effect in the network.

More in details, NBS works as follows. The first step involves calculating an individual univariate test statistics for each connection, as e.g., the *t*-test statistics. After that, a threshold defined by the user is applied, resulting in a set of supra-threshold links. This threshold is the minimal value of the test statistics of an edge that can potentially reject the null hypothesis. The following step is to use a breadth-first search algorithm on the set of links, to find the number of resulting connected components and their sizes. These components can be considered as subnetworks containing connections that have a statistical effect. The size of the largest component is saved; additionally, and using repeated permutation tests, the members of the samples are randomly permuted, and the size of the largest component is stored. Finally, the edges of the final component are retained for further analyses. NBS comes with a gain in statistical power, but its weakness is that one cannot localise the effect to individual edges, as instead complete connected components are retained.

Several improvements and variations of the NBS testing procedure have been proposed. To illustrate, a threshold-free variant is described in [18]. A specific procedure was proposed to adjust the cluster sizes in [19], and implemented in the permutation-testing framework in the Mrtrix connectomestats software (https://mrtrix.readthedocs.io/en/latest/reference/commands/connectomestats.html, accessed on 20 February 2021). Finally, other highly data adaptive tests that choose several parameters to reflect uncertainty with gained power are proposed in [20].

We used the basic NBS implementation available at https://github.com/aestrivex/bctpy/blob/master/bct/nbs.py (accessed on 20 February 2021), with a grid search to find the optimal threshold that leads to the best classification.

#### 2.2.3. Adaptive Sum of Powered Score Test

The sum of powered score test, also known as the SPU test, was initially proposed in [21] for identifying rare variants in genome-wide association studies, and has subsequently been used on brain networks [22]. This approach provides a global testing, i.e., the significant effect cannot be associated to individual edges.

The simplest SPU test computes a score vector *U* as $U=\sum_{i=1}^{n}X_{i}\left(Y_{i}-\hat{Y_{i}}\right)$, where $X_{i}$ are the observations (brain network half-vectorized) for the *i*-th individual, *Y* is a binary vector indicating the group of the individuals, and $\hat{Y}$ the mean of the latter. Each element $U_{j}$ contains the non-zero effect of each connection in the network. This score vector is used to compute the test statistics as $T_{SPU}=\sum_{j=1}^{k}U_{j}^{\gamma }$, where γ is an input parameter that should be tuned in order to obtain the highest statistical power. To make a statistical inference, the next step is to perform parametric bootstrapping or permutations, obtaining corrected *p*-values.

The adaptive sum of powered score test (aSPU) is a variation aimed at finding the optimal value of the parameter γ [21]. If Γ=1,2,…,∞ and $P_{SPU}\left(\gamma \right)$ is the *p*-value of the SPU test with parameter γ, then the statistics for the aSPU test is computed as $T_{aSPU}=\min_{\begin{array}{c} \gamma \in \Gamma \end{array}}P_{SPU}\left(\gamma \right)$. The *p*-value of the test statistics is calculated through bootstrapping or permutations. [21] demonstrates the use of the score component $U_{j}$ as the contribution of each connection to the test statistics. One can thus order connections according to their $U_{j}$, and select the top-*k* as the *k* most important ones. We used the R implementation of the basic aSPU test available at https://github.com/ikwak2/aSPU (accessed on 25 February 2021).

#### 2.2.4. Two Sample HOTELLING’s T-Square Statistics

Hotelling’s $T^{2}$ statistics [23,24] is a natural multidimensional generalisation of Student’s t statistics. This test compares the mean values of the samples containing data with multiple variables (characteristics). In order to perform a link selection, one starts by calculating *N* Laplacian matrices, one per subject, as L=D−A, where *D* and *A* are respectively the corresponding degree and adjacency matrices. Afterwards, two Fréchet means are calculated, of respectively the Laplacian matrices of the two groups of subjects; these mean matrices are then transformed into vectors through half-vectorization, and are denoted in what follows by $vec({\hat{L}}_{1})$ and $vec({\hat{L}}_{2})$.

The Hotelling’s two-sample T-Square statistics is then defined as

(1) $$T^{2}=\frac{n_{1}n_{2}}{n_{1}+n_{2}}\left({vec(\hat{L}}_{1}\right)-{vec(\hat{L}}_{2}){)}^{T}{\hat{\Sigma }}^{-1}\left({vec(\hat{L}}_{1}\right)-{vec(\hat{L}}_{2})),$$

where ${\hat{\Sigma }}^{-1}$ is the inverse of the covariance matrix $\hat{\Sigma }=\frac{1}{n_{1}+n_{2}-2}\sum_{j=1}^{2}n_{j}{\hat{\Sigma }}_{j}$, with ${\hat{\Sigma }}_{j}$ denoting the individual sample covariance matrix

(2) $$\hat{\Sigma }=\frac{1}{n-1}\sum_{i=1}^{n}\left(vec\left(L_{i}\right)-vec\left(\hat{L}\right)\right){\left(vec\left(L_{i}\right)-vec\left(\hat{L}\right)\right)}^{T}$$

and $n_{1}$ and $n_{2}$ the samples sizes. According to [24], when the sample size is big enough, $T^{2}$ is approximately chi-square distributed with *p* degrees of freedom. When the hypothesis of normality holds, the Hotelling test is optimal.

With this definition of the $T^{2}$ statistic, the next step is to calculate the covariance matrix. As noted in [25], the sample (empirical) covariance based on the observed data is singular when the dimension is larger than the sample size (n<<p), i.e., it cannot be inverted to compute the precision matrix. Also, the sample covariance matrix is likely to be numerically unstable. Therefore, an estimation method for obtaining a proper covariance matrix should be used. Multiple estimators have been developed as a solution to the problem and they are applied in various fields [26]. A part of them are estimators of covariance matrices [26], some are for sparse covariance matrices [25,27], and others are used to estimate the inverse of the covariance matrix [28]. We here use a form of shrinkage estimation [29] that shrinks the sample covariance matrix towards the identity matrix, and which is equivalent to considering an asymptotically optimal convex linear combination of the two. The implementation corresponds to the one in [30]. We then calculate the covariance matrix from the individual estimated covariance matrices, the $T^{2}$ statistics, the degrees of freedom, and *p*-value. Using *p*-value < α, we determine whether the samples are different.

The contribution of each edge to the $T^{2}$ statistic can be determined through a linear decomposition of $T^{2}$. By taking the square root of the inverse covariance matrix, one can make the decomposition $\frac{n_{1}+n_{2}}{n_{1}n_{2}}T^{2}=A^{T}A=\sum_{i=1}^{p}A_{i}^{2}$, where $A={\hat{\Sigma }}^{-\frac{1}{2}}\left({vec(\hat{L}}_{1}\right)-{vec(\hat{L}}_{2}))$, and $A_{i}^{2}$ is the contribution of the $i^{th}$ edge to the value of $T^{2}$. With this, the inverse matrix is decomposed through spectral decomposition, and the weights for the edges are estimated. Finally, edges are sorted according to their weight, and the top-*k* edges are retained.

#### 2.2.5. Difference Degree Test (DDT)

The difference degree test (DDT) [31] is a method for brain networks comparison proposed to overcome the low statistical power of mass-univariate approaches. The test finds the difference degree network that identifies the brain regions connected to a significant number of differentially weighted edges (DWEs).

The first step of the test involves constructing the difference network $D=d_{ij}$, i,j∈1,2,…,N; each element $d_{ij}$ represents the statistical difference in the connections between nodes *i* and *j*, and is defined as the *p*-value of a between group difference test. Afterwards, the first and second moments of the difference network *D* are obtained as $\bar{e}=E\left({\bar{d}}_{ij}\right)$ and $\sigma^{2}=Var\left({\bar{d}}_{ij}\right)$, according to which a certain number of random networks are generated using the efficient algorithm proposed in [32]. The random networks are thus matched to the observed difference network, to capture its true topology. Afterwards, an adaptive threshold selection method is applied for thresholding *D*. In our analysis, we used the empirical difference degree test (eDDT), which uses the empirical critical value for thresholding. The selected threshold is applied to *D* and the difference adjacency matrix $A=a_{ij}$ is obtained, where $a_{ij}$ represents the differences in the connection between nodes *i* and *j*. Based on *A*, a difference degree measure $d_{i}$ is calculated for each node *i*. $d_{i}$ is modeled with a binomial distribution $B(N-1,p_{i}^{null})$, where $p_{i}^{null}$ is the expected probability for each connection of node *i* to demonstrate between-group differences and it is computed based on the generated random networks. Finally, the statistical significance of the DWEs at each node is assessed. We here propose to create the list of relevant links by selecting those that are connected to a statistically significant difference node.

#### 2.2.6. Anova Networks Test (AnovaNet)

Another newly developed statistical test for identifying differences in populations of networks is the analysis of variance (ANOVA) networks (AnovaNet) test [33]. It aims at identifying subnetworks where different types of modifications between groups occur: modifications localized just in particular edges; unlocalized modifications, in which some edges are changed but they differ among individuals; and global modifications, in which there are global alterations among groups. As a result, the algorithm yields a subset of edges, or a subnetwork, where the highest network difference between groups is present.

Given the complete set of brain networks $G_{1},G_{2},\ldots ,G_{n}$, the AnovaNet test compares two groups by calculating the statistic $T=\frac{\sqrt{2}}{a}\sum_{i=1}^{2}\sqrt{n_{i}}\left(\frac{n_{i}}{n_{i-1}}{\bar{d}}_{G^{i}}\left(M_{i}\right)-\frac{n}{n-1}{\bar{d}}_{G}\left(M_{i}\right)\right)$, where $n_{i}$ (i∈1,2) are the number of networks in each group, $n=n_{1}+n_{2}$, *a* is a normalization constant, ${\bar{d}}_{G}\left(M\right)=\frac{1}{l}\sum_{k=1}^{l}d(G_{k},M)$ is the average distance around a network (calculated using the edit distance between graphs [34]) and $M_{i}$ (i∈1,2) is the average adjacency matrix for group *i*. In other words, this statistic compares the network variability for each group on one hand, and the average distance between networks of one group and their corresponding average on the other.

As a last step, an additional statistic $T_{\bar{E}}$ is defined, as the T statistic obtained for the subnetwork composed by all nodes and a subset $\bar{E}$ of all edges. The edges $\bar{E}$ that minimises $T_{\bar{E}}$ are the ones most contributing to the difference among the groups, and are therefore retained for subsequent analyses.

#### 2.2.7. NEtwork-VAlued Data Analysis (Nevada)

The model-free two-sample test for network-valued data (Nevada) [35] is a permutation test for making inference on distributions of networks. The proposed framework is very flexible, since it allows choosing different matrix representations of the networks, different distance metrics and different test statistics.

Nevada can be adapted to perform link selection as follows. The first step involves choosing the matrix representation, among the several available, that gives the best results: adjacency, Laplacian or modularity matrices. Secondly, one must choose a statistic; a large number of them are reviewed, proposed and used in [35]. Given the two groups i=1,2 and the corresponding networks $G_{i}=${$G_{i1},G_{i2},\ldots ,G_{in_{i}}$}, we use the T statistic to detect higher-moment differences between networks defined as $T=\frac{\frac{1}{n_{1}n_{2}}\sum_{i=1}^{n_{1}}\sum_{j=1}^{n_{2}}d^{2}\left(G_{1i},G_{2j}\right)-\left(\hat{\sigma_{1}^{2}}+\hat{\sigma_{2}^{2}}\right)}{\frac{\hat{\sigma_{1}^{2}}}{n_{1}}+\frac{\hat{\sigma_{2}^{2}}}{n_{2}}}.$ Here, $\hat{\sigma_{1}^{2}}$ and $\hat{\sigma_{2}^{2}}$ are estimators of the within-sample variances in the two groups, and are defined as $\hat{\sigma_{1}^{2}}=\frac{1}{n_{1}\left(n_{1}-1\right)}\sum_{i=1}^{n_{1}}\sum_{j>i}^{n_{1}}d^{2}\left(G_{1i},G_{1j}\right)$ and $\hat{\sigma_{2}^{2}}=\frac{1}{n_{2}\left(n_{2}-1\right)}\sum_{i=1}^{n_{2}}\sum_{j>i}^{n_{2}}d^{2}\left(G_{2i},G_{2j}\right)$. Note that the distance $d(G_{k},G_{l})$ between two networks $G_{k}$ and $G_{l}$ is here defined as the corresponding Frobenius distance.

As a final step, a permutation test is designed with a non-parametric combination approach that captures both mean and variance differences between networks groups with high statistical power. Similarly to the AnovaNet approach, the edge subset that yields the optimal value of the statistic, and therefore the largest differences between the two groups, is selected. In our implementation we used the nevada (NEtwork-VAlued Data Analysis) R package taken from https://github.com/astamm/nevada (accessed on 25 February 2021).

It is worth noting that other statistical methods for detecting group differences in network structures have been proposed - the interested reader may refer to [36,37,38,39,40]. These methods have not been included here due to practical and implementation issues, as e.g., too large computational costs.

### 2.3. Machine Learning

Feature selection is an active field of research within the machine learning community, and as such a large number of algorithms and methods have been proposed. While in principle all of them could be applied to functional brain networks, a complete review goes beyond the scope of this contribution—review that the interested reader can find, for instance, in [41,42]. Here, nine of the most representative and well-known methods are instead discussed and evaluated. For the sake of clarity, there are further divided into three families, following the customary classification in machine learning: filtering, wrapper and embedded methods [41].

#### 2.3.1. Filtering Methods

Feature filtering methods use certain metrics to rank features and to form the best set of attributes [43]. They are independent of the learning algorithm, and therefore are most often used as a pre-processing technique. Filtering methods determine the relevance of features by correlating them with the dependent variable, using metrics like information gain, Gini index and others [44,45,46].

#### Spatially Uniform Relief (SURF)

Relief [47] is an feature ranking method based on instances for a binary classification problem. ReliefF [48] is an extension of Relief for multi-class problems. Relief ranks features depending on how well they differentiate between instances that are close to each other.

The algorithm randomly selects an instance $G_{i}$ from the data and finds the nearest neighbor *H* from the same class and the nearest neighbor *M* from another class. It then updates the feature score by comparing the values of the features of $G_{i}$ with *H* and *M*. If $G_{i}$ and *H* have different values for the feature *f*, i.e., the two instances of the same class are separated by *f* (which is not desirable), the rank score of *f* is decreased. On the other hand, if the feature *f* separates $G_{i}$ and *M* (which is desirable), then the *f* rank score is increased. The process is repeated for *m* randomly selected instances. ReliefF is also more robust than Relief using *k* nearest neighbors. ReliefF ranks all features and needs a predefined threshold *t* for choosing the best *N* features.

The SURF algorithm [49] builds on top of ReliefF, but, unlike this, eliminates the user parameter *k* and instead adopts a threshold at a distance *T* to determine which cases will be considered neighbors. We have used the SURF implementation included in the scikit-rebate package in Python, and available at https://github.com/EpistasisLab/scikit-rebate (accessed on 26 February 2021).

#### 2.3.2. Wrapper Methods

Wrapper methods use a set of features to learn a model, for then using the parameters of the model to evaluate the selection of features. In other words, they decide whether to add or remove features from the subset by evaluating the impact that those features have in the classification score. The problem is essentially reduced to a search one, making it usually computationally very expensive. Additionally, there is an increased risk of overfitting, especially when the number of observations is low. Well-known examples of wrapper methods are forward feature selection, backward feature elimination, and recursive feature elimination. We use the recursive elimination approach using the SVM learning model (SVM-RFE), and logistic regression (LR-RFE), as well as Boruta.

#### Support Vector Machine-Recursive Feature Elimination (SVM-RFE)

This method, called support vector machine-recursive feature elimination (SVM-RFE), is based on performing a recursive feature elimination (in which features are deleted one at the time) under the guidance of a linear SVM classifier [50]. The SVM algorithm divides a given set of instances using a maximum margin hyperplane, i.e., an hyperplane that is maximally separated from the two classes of instances. The objective function is to find a division function that will accurately classify new samples. The parameters of a linear SVM define a decision hyperplane in the multidimensional feature space, i.e., $g\left(x\right)=w^{T}x+b=0$, where *x* is the features vector, *w* is the vector with weights for the features, and *b* is a bias parameter.

According to the SVM-RFE algorithm, the relevance of the *n* element in the feature vector is determined by the value $w_{n}$ from the weight vector. Practically, if $w_{n}$ is close to 0 the corresponding feature does not contribute significantly to the value of g(x). The importance of each feature is then determined by sorting the absolute values of the vector *w*, and, in each iteration of the algorithm, a specific number of features are discarded.

#### Logistic Regression with Recursive Feature Elimination (LR-RFE)

The logistic regression (LR) model predicts the parameters of a logistics model in order to model class probabilities. Each feature of the training data is associated to a number between 0 and 1, which represents the probability of belonging to a given class. The basic idea of logistic regression is to use the mechanism already developed for linear regression by modeling the probability $p_{i}$ using a linear prediction function, i.e., a linear combination of given attributes and a set of regression coefficients. The linear prediction function *f* is given by f(x)=w∗x+b=log(p(x)/(1−p(x)), where *w* the vector with the regression coefficients, *x* is a vector of predefined data attributes, and *b* refers to the bias variable. In a manner similar to the SVM-RFE approach, we use a recursive feature elimination algorithm to determine the importance of each feature, sort them and take a specific number of features (here, links) as significant.

#### Boruta

The Boruta [51] algorithm is a wrapper feature selection method around the Random Forest classification algorithm. The algorithm first duplicates the set and shifts the features values, which are called shadow features, in the newly created one. The next step involves training a Random Forest algorithm to merge the original and the modified feature sets. The values for feature significance are taken from the trained model. The algorithm then checks which of the real features were more important than the modified features. The random shift of the features, the learning of the algorithm, as well as the checking of the significance of the real features is repeated *N* times and for each feature the number of hits is calculated (number of iterations in which that feature had a significance greater than the maximum significance of the modified features). A statistical test is performed in which the feature is rejected if the number of hits is less than the expected value, and it is retained otherwise. In the implementation, a two-step correction method is used to correct the error that occurs during multiple testing. First, an FDR correction is used, and then a Bonferroni correction to capture that the same features are tested over and over again in each iteration with the same test.

We use the implementation of this algorithm in Python available at https://github.com/scikit-learn-contrib/boruta_py (accessed on 26 February 2021), which has modifications to loosen the strictly defined rules in the original code in R, specifically, it introduces a percentile to substitute the fixed rejection threshold.

#### Sparse Linear Discriminant Analysis (SLDA)

A Sparse Linear Discriminant Analysis (SLDA) feature selection process for brain networks is proposed in [52], based on two stages, and using a machine learning technique that includes sparsity for weighted networks. In the first stage of the feature selection, a sparse linear discriminant analysis is performed to select discriminant features. Different subsets of the brain networks dataset are iterated with a multivariate bootstrap approach. Moreover, also in the first step, an ensemble of sparse linear discriminant analysis (LDA) models is constructed, using the sparsity idea from [53] to find the features (network links) that discriminate the two brain network groups. The subset of the selected features is minimized, and the difference between the two groups is maximized. The second step is a stability selection one, where only the features that are frequently selected across iterations are kept.

The definition of the sparse LDA problem uses l1 and l2 regularizations as in a Elastic-net problem (see below). The l1 regularization ensures a reduction in the overfitting, by forcing the choice of a small number of features. Furthermore, the l2 regularization minimizes the prediction error and helps managing the high-dimensionality of the data. The two combined give an optimal classifier and a minimum number of discriminant features.

For our implementation we have used an R alternative to the original Matlab code (https://github.com/alecrimi/multi-link, accessed on 27 February 2021) taken from https://github.com/topepo/sparselda (accessed on 27 February 2021).

#### 2.3.3. Embedded Methods

Embedded methods combine the qualities of filtering and wrapper methods. They are implemented with algorithms that have their own built-in feature selection methods. The learning algorithm utilizes its own feature selection process and at the same time performs feature selection and classification/learning.

#### Lasso

The Least Absolute Shrinkage and Selection Operator (Lasso) is a regression analysis method that includes feature selection and regularization in order to improve the accuracy and interpretation of the obtained statistical model [54]. Lasso is able to achieve these two goals by setting a fixed value for which the sum of the absolute values of the regression coefficients must be minimized, forcing certain coefficients to be set to zero, effectively choosing a simpler model that does not include irrelevant coefficients. The used implementation is based on a l1 regularization in a logistic regression in Python.

#### Elastic-Net

Another well-known way of adding regularization is the Ridge method (l2 regularization), which uses a square instead of an absolute value as in Lasso. The method known as Elastic-Net [55] uses the Ridge and Lasso methods simultaneously: $L+\lambda (\left(1-\alpha \right)\sum w^{2}+\alpha \sum |w|)$, where α controls the mix of Ridge and Lasso regulations, with α=0 value indicating “pure” Ridge, and α=1 “pure” Lasso. Our implementation uses the SGDClassifier from the scikit-learn package [30] in Python, with an elastic-net regularization α=0.15.

#### Random Forest (RF)

The random forest (RF) classification algorithm has a built-in feature importance computation, capturing the features’ contribution in decreasing the weighted impurity of a split while training the trees composing it. Features are selected as internal node in the tree according to their impurity decrease, which, in the case of classification tasks, can be computed through Gini impurity or infomation gain. The average of this decrease of each feature in all trees of the forest is the measure for feature importance. We select the top *k* features (edges) with the highest importance using the implementation of RF classifier from scikit-learn package in Python.

#### Extra Trees (ExT)

In a way similar to what done in the previous RF method, we use the built-in feature ranking included in Extremely Randomized Trees Classifiers (Extra Trees). Extra Trees is an ensemble learning method very similar to random forest, different only in the way the decision trees are constructed. For performing the feature selection, during the construction of the forest, for each feature, a normalized total decrease in the criteria used in the decision of the split (usually a Gini index) is calculated as feature importance. The features are sorted in descending order of their importance and the top *k* features are retained. Similarly to the previous methods, we used a Python scikit-learn implementation with Extra Trees classifier.

## 3. Datasets and Classification Models

### 3.1. Brain Image Acquisition and Network Reconstruction

#### 3.1.1. COBRE

The first dataset used in this study is the Schizophrenia dataset COBRE [56], which includes Schizophrenia patients (SZ) and healthy control (HC) subjects. We have selected 144 subjects (70 SZ and 74 HC), excluding two subjects from the original dataset for being outliers. A 3T Siemens Trio scanner was used to acquire anatomical multi-echo MPRAGE sequence data (TR/TE/TI = 2530/[1.64, 3.5, 5.36, 7.22, 9.08]/900 ms, FOV = 256 × 256, FA = 7°, voxel size = 1 × 1 × 1 mm${}^{3}$ [56]) and rs-fMRI data (TR = 2 s, TE = 29 ms, FA = 75°; FOV = 240 mm, matrix size = 64 × 64, voxel size = 3.75 × 3.75 × 4.55 mm${}^{3}$ [56]). We did fMRI preprocessing using the Statistical Parametric Mapping (SPM12) [57] library for MATLAB R2018b and the CONN toolbox [58] version 18b. A default pipeline was used with standard preprocessing steps: realignment and unwrap; slice-timing correction; outliers detection of functional volumes using ART [59]; segmentation of gray matter, white matter, and cerebrospinal fluid (CSF) areas for the removal of temporal confounding factors; normalization of images to the standard Montreal Neurological Institute (MNI) template; smoothing with an 8mm full width at half maximum (FWHM) kernel); and band-pass filtering with a frequency window of 0.008–0.09 Hz. Finally, to construct the 116 × 116 functional connectivity matrices using Fisher-transformed bivariate correlation in the CONN toolbox, the Anatomical Atlas Labeling (AAL) [60] template was used for brain ROI parcellation.

#### 3.1.2. PRURIM

The second dataset here used is taken from the USC Multimodal Connectivity Database [61] http://umcd.humanconnectomeproject.org (accessed on 15 February 2021), which is a web-based brain network platform and database. It includes 29 functional 116 × 116 connectivity matrices coming from a study with 14 patients with chronic psoriasis and 15 healthy controls [62]. The fMRI imaging data come from the University Hospital Brest (CHU de Brest), France, acquired with a Philips Achieva dStream 3T scanner (fMRI data with TR = 3.15 s, First Echo Time = 17 ms, Second Echo time = 46 ms, voxel size = 2.5 × 2.5 × 2.6 mm${}^{3}$, FA = 90°, FOV = 250 × 250 × 143, matrix size = 128 × 128, number of slices = 45). Standard pre-processing steps (slice time correction, realignment, coregistration, normalization, smoothing with 10 mm FWHM, parcellation with AAL atlas, regressing out confound factors, and band-pass filtering) have been performed and 29 functional connectivity matrices have been uploaded to the USC Multimodal Connectivity Database.

#### 3.1.3. UCSF Progressive Supranuclear Palsy (PSP)

The third dataset here used is also taken from the USC Multimodal Connectivity Database [61]. It includes 64 connectivity matrices with size 27 × 27 from a task-free functional magnetic resonance imaging (tf-fMRI) study of 12 Progressive Supranuclear Palsy (PSP) patients and 20 healthy controls [63,64] scanned twice 6 months apart. They are constructed from imaging data from the UCSF Neuroscience Imaging Center, San Francisco, acquired with a Siemens Trio 3T scanner (tf-fMRI data with acquisition time = 8 min 6 s, axial orientation with interleaved ordering, FOV = 230 × 230 × 129, matrix size = 92 × 92, voxel size = 2.5 × 2.5 × 3.0 mm${}^{3}$, TR = 2000 ms, TE = 27 ms). Additional information regarding the preprocessing steps and the reconstruction of the connectivity matrices is available in [63].

### 3.2. Classification Algorithms

Once multiple sets of features, i.e., of links, are obtained through the different methods described in Section 2, these are evaluated through a classification problem. In other words, a classification model is trained to discriminate between the two groups of people represented in the datasets, only using the selected links, and the result is evaluated in terms of the Area Under the Curve (AUC) and F1 metrics. These are metrics that assess the performance of a classifier, where the AUC represents a degree for separability of classes, and the F1 score is a measure that balances between precision and recall of the model. Intuitively, the higher the obtained classification score, the more information relevant for the classification problem is included in the used set of links; consequently, the classification score can be used as a proxy of the effectiveness of each link selection method [65].

As different models are based on different assumptions on the features and their relationships, each classification has been performed using multiple algorithms with repeated stratified 10-fold cross-validation for validation, in order to avoid model-specific biases. There are:

- Bayes: Also known as naïve Bayes classifier, this model assumes complete independence between the input features of the problem, for then directly applying the Bayes theorem and estimate the probability associated to each class [66]. While being highly scalable and of low computational cost, its main limitation is the independence assumption, as it cannot usually be guaranteed.
- Gaussian Naïve Bayes (GNB): A type of naïve Bayes classifier in which input features are considered to be continuous (as opposed to discrete) and modelled as a normal (or Gaussian) distribution.
- Decision Trees (DT): Classification models composed of a tree-like structure of nodes. Each node represents an attribute (feature) in a record to be classified, which is tested against a question defined in the training phase; each branch then represents a value that the attribute can take, i.e., an answer to the node’s question. The DT’s main advantage resides in its simplicity and reduced computational cost [67].
- Random Forest (RF): Combinations of Decision Tree predictors, in which each tree is trained over a random subset of features and records; the final classification forecast is then calculated through a majority rule. Random Forests are especially appreciated for their precision and low tendency to overfitting [68].
- ExtraTrees (ExT): an ensemble fast method based on the combination of a number of randomised decision trees on various sub-samples of the dataset, in a way similar to the Random Forest classifier. Improvement of the predictive accuracy and control of the over-fitting are gained by adding randomisation in choosing the optimum cut split points [69].
- Stochastic Gradient Descent (SGD): meta-algorithm in which multiple linear Huber loss functions are combined and optimised [70].
- Ada: a meta-algorithm whose output is the weighted sum of a large set of small decision trees, where the weights are dynamically adjusted to minimise the classification error [71].
- AdaBoost: an iterative ensemble method [72] that combines multiple weak base classifiers to obtain a final model of higher accuracy. It iteratively fits copies of the base classifier with adjusted weights, such that subsequent classifiers are focused on unusual observations.
- Multi-Layer Perceptron (MLP): based on the structural aspects of biological neural networks, MLPs are composed of a set of connected nodes organised in layers. Each connection has a weight associated to it, which is tuned through the learning phase [73]. When more than two layers are included in the model, it can be proven that MLPs can classify data that are not linearly separable, and in general approximate any non-linear function.
- Support Vector Machine (SVM): model that learns a classification by finding the best curve, in the hyper-space created by the features, for dividing the instances according to their class, such that instances laying in one of the two parts of the hyper-space are mostly of one class [74]. We consider two alternatives: a linear curve, thus dividing the hyper-space with an hyper-plane (LinSVM); and a Radial Basis Function kernel, based on the use of euclidean distances, and thus separating instances using hyper-spheres (RBFSVM).
- Ridge: model that learns a classification rule by calculating a ridge regression between the input features and the known classes. The ridge regression is similar to the standard linear one, but includes a penalty on the size of the coefficients, yielding a more robust solution in the presence of collinearities [75].
- Gradient Boost (GradBoost): classification model composed of a large set of weak classifiers, in this case DTs. The main difference with respect to RF is that here trees are added one at the time, using a gradient descent-like procedure, and previously added trees are frozen and left unchanged [76].
- Logistic Regression (LR): a logistic regression model used as a (generally effective) classifier. It takes a linear equation as input and uses a logistic function to perform a binary classification task [77].
- K-Nearest Neighbours (KNN): simple classification model in which the class of a new instance is set as the class most common among its *k* nearest neighbours. While it is one of the most efficient algorithms and requires no initial training, it is also sensitive to the local structure of the data, and may not be suitable for all problems [78].
- Voting: model based on combining multiple classifiers to make predictions based on the most frequent one according a voting procedure [79]. It is especially well-suited in situations where the best classification method is not known.
- Bagging: an ensemble method that builds several random instances of a base estimator (here, decision trees) on random subsets of the original training set. Individual predictions are aggregated in a final prediction. In this way the variance of the base estimator is reduced, and stability and accuracy improved [80].
- XGBoost: variant of the Gradient Boost algorithm optimised for processing large data sets, by leveraging both on parallel and distributed computation [81].

Some of the classification models have one or more parameters that require an initial tuning. In those situation, we have considered both a naïve solution, leaving those parameters to their default values; and a grid search procedure, which determines the optimal hyperparameters of the model by maximising the final classification score.

We finally consider two additional models, based on the concept of Deep Learning (DL). DL encompasses machine learning algorithms that progressively extract higher-level features from the raw input, usually with the objective of performing a supervised classification [82,83]. They present the advantage of achieving high precision, although at the cost of requiring large training sets, of high computational costs, and of being black boxes, i.e., they don’t facilitate recovering the logic behind a result. The first model is a basic approach with a fully connected deep neural network (DNN) with 3 layers, 2 ReLU and 1 Sigmoid. The second model is a deep graph convolutional neural network (DGCNN) [84,85] with the significant edges extracted from the graph from the feature selection process as input.

## 4. Results

As initially introduced, the link selection methods described in Section 2 are here evaluated against the COBRE dataset according to three main criteria: their performance, in terms of quantity of retained information (Section 4.1); their stability, i.e., how consistently they yield the same set of links (Section 4.2); and their computational cost (Section 4.3). These results are then compared in Section 4.4 with those obtained for the two remaining datasets, PRURIM and PSP, to assess their generalisability.

### 4.1. Performance Comparison

**Figure 2:**
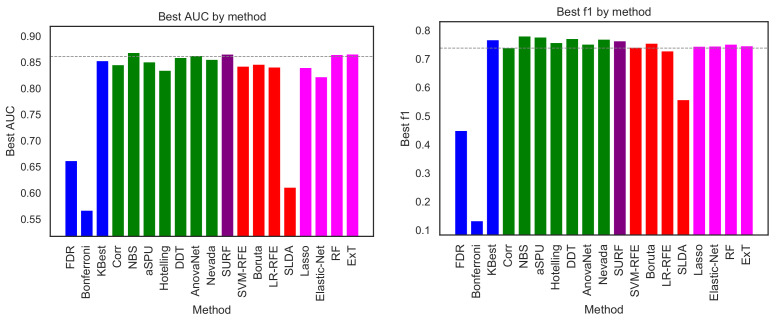
Best classification score, in terms of AUC (**left panel**) and F1 (**right panel**), obtained by the 19 feature selection methods here considered. Bar colours indicate the family of each method, using the same code as in Figure 1. The horizontal dashed line represents the classification score obtained by a RF model on the whole connectivity matrix, i.e., without any link selection.

As a first analysis, Figure 2 depicts the best classification obtained by each selection method, measured in terms of AUC (left panel) and F1-measure (right panel). For each selection model, the maximum has been calculated across all classification models. In other words, what depicted in Figure 2 is how good the classification is, provided the best classification algorithm is known and used in each case. The resulting classification score is then used as a measure of the quantity of information retained by each link selection method.

Results are generally quite homogeneous. Excluding three outliers (SLDA, FDR and Bonferroni), the 16 remaining methods are included between NBS (AUC of 0.869) and Elastic-Net (AUC of 0.822). When comparing methods using the F1-measure, a similar picture arises, with the 16 methods laying between 0.780 (NBS) and 0.728 (LR-RFE). This suggests that the choice of a specific selection method has a minor impact in the final classification; and, accordingly, that the 16 methods are all effective in retaining relevant information about the studied condition. This of course does not hold in the case of SLDA, FDR and Bonferroni, which clearly perform worse than the others; and, in the case of Bonferroni, even approach the barrier of what expected in a random classification. It is important to highlight that the classification scores here reported are the average across a 10-fold cross-validation; when the standard deviation of these executions is taken into account, respectively 0.094 for the NBS and 0.085 for the Elastic-Net (both for AUC), it becomes clear that the difference between the best and worst algorithm is negligible. To further put these numbers in context, the horizontal dashed lines of Figure 2 report the classification obtained by a Random Forest algorithm using the complete connectivity matrices. These results, respectively 0.861 for the AUC and 0.738 for the F1-measure, indicate that discarding non-informative links is not only retaining all relevant information, but could also be beneficial in a classification problem—an effect that is well-known in machine learning.

**Figure 3:**
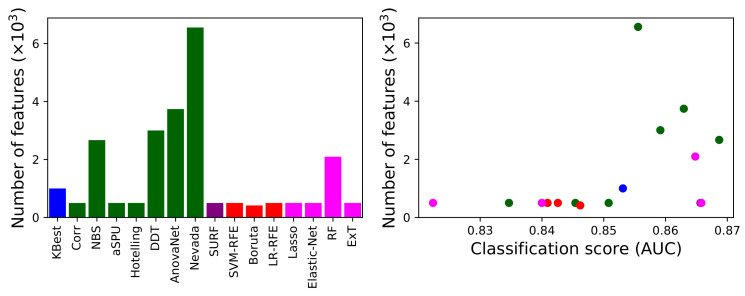
(**Left**) Number of features retained by each method (excluding the three underperforming ones), in thousands. (**Right**) Number of retained features as a function of the score (AUC) achieved by each method. The colour of each method corresponds to the one in Figure 1.

We next analyse how the methods behave in terms of the number of links they recommend for the analysis. Figure 3, left panel, depicts such number; and the right panel presents a scatter plot of this number as a function of the classification score (AUC). Note that three underperforming methods, according to Figure 2, have been omitted in Figure 3 for the sake of clarity. It can be appreciated that, as expected, methods selecting few features are generally the ones performing worse. While the opposite is generally true, it is worth noting that the method yielding the highest number of features (i.e., Nevada) is not the best performing one (NBS, as also previously seen).

**Figure 4:**
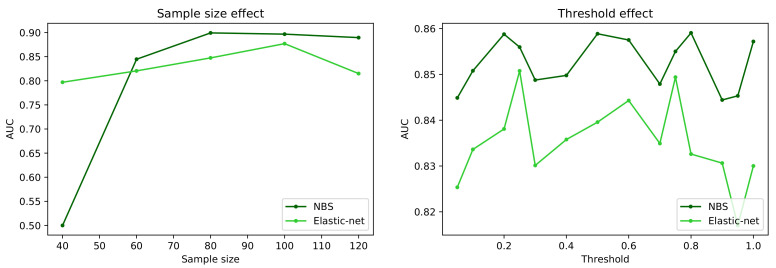
Evolution of the classification score, in terms of AUC, as a function of the sample size (number of subjects in the dataset, (**left panel**)) and of the proportional threshold applied to the network (**right panel**). Black and green lines respectively correspond to the NBS and Elastic-net link selection methods.

Figure 4 reports a comparison of the behaviour of the best and worst performing methods, i.e., respectively NBS and Elastic-Net, when two important characteristics of the dataset are synthetically changed: the number of subjects in it (left panel); and the way networks are preprocessed, and specifically when a given fraction of the strongest links are retained, and all others are deleted (a process known as proportional thresholding [86], right panel). In both cases, the classification task has been executed with the machine learning model yielding the best result, i.e., the model yielding the score of Figure 2. As is to be expected, reducing the sample size has a negative effect on the classification score of both algorithms, although the behaviour is uneven. Elastic-net seems to perform better with small data sets, with an almost linear decrease in the AUC, as opposed to a transition to random classifications (i.e., AUC ≈0.5) displayed by NBS for sample sizes smaller than 60 subjects. The right panel further suggests that the initial link deletion step has a negligible impact in the output of both algorithms. Specifically, changing the threshold does not change the ranking of the two methods, nor significantly change the AUC. Note that the difference between the maximum and minimum AUC for each method is <0.04, i.e., smaller than the standard deviation observed in the cross-validation process; the observed fluctuations are thus the result of the stochasticity of the classification models.

### 4.2. Intra- and Inter-Method Stability

When dealing with different feature selection methods, two important aspects are the intra- and inter-method stability. More in details, one wants to assess whether a given method always yields the same set of features for a fixed problem (here called intra-method stability); and whether two different methods yield sets of features that are compatible, or overlapping (inter-method stability). Note that an unstable feature selection can lead to inferior classification performance; but, more importantly, it can undermine any attempt at understanding the mechanisms involved in a condition or pathology.

In order to assess both stabilities, we here use the similarity method proposed in [87]. The similarity of two selected sets *s* and $s^{\prime }$ is calculated as one minus the Tanimoto distance, which measures the overlap of two sets with arbitrary cardinality:

(3) $$D\left(s,s^{\prime }\right)=1-\frac{|s|+|s^{\prime }|-2|s\cap s^{\prime }|}{|s|+|s^{\prime }|-|s\cap s^{\prime }|}.$$

This metric has a value in range [0,1], where 0 means that there is no overlap or similarity between two feature rankings, and 1 means that the feature rankings are identical.

**Figure 5:**
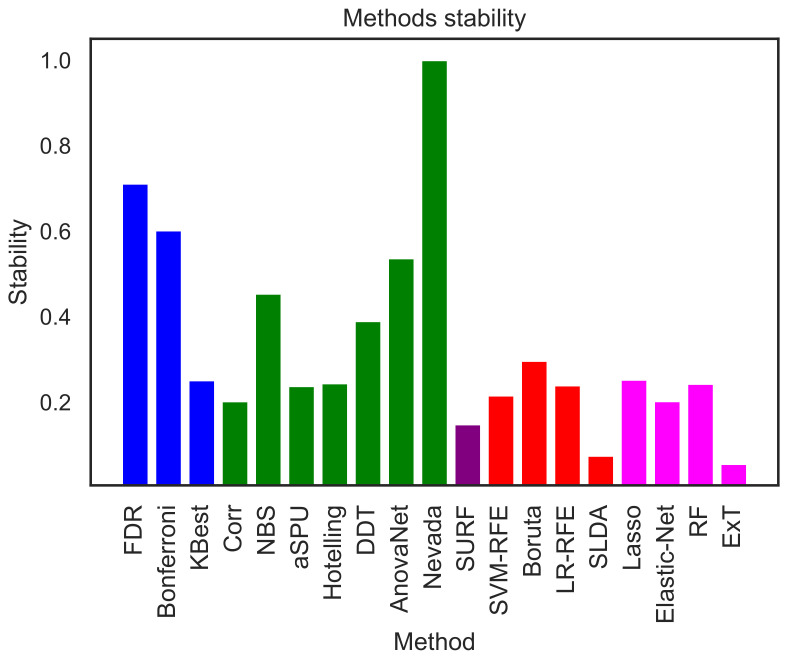
Intra-stability *D* of each method, representing how consistently each method is yielding the same link selection over random subsamples of the original dataset. See Equation (3) for a definition of the metric *D*.

Figure 5 reports the intra-stability of each selection method. For each one of them, 100 subsets comprising all the features but only 100 subjects, selected from the original set using random resampling via bootstrapping, have been constructed; the displayed value corresponds to the average over all pairwise values of *D*. Nevada stands out as the method with the higher intra-stability; this is nevertheless not surprising, as this was also the method yielding the larger number of elements—see Figure 3. After it, the two most stable methods are FDR and Bonferroni; yet, these were two of the underperforming ones identified in Figure 2, suggesting that they are consistently selecting the wrong interactions. Finally, the two following ones are AnovaNet and NBS, corresponding to the two best performing methods.

**Figure 6:**
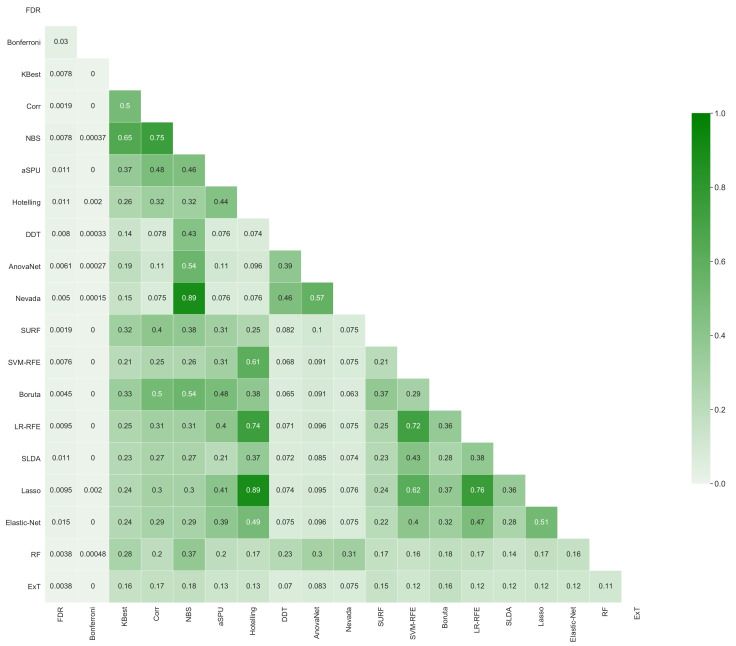
Inter-stability of each pairs of methods, i.e., fraction of common links they both yield, measured according to Equation (3).

**Figure 7:**
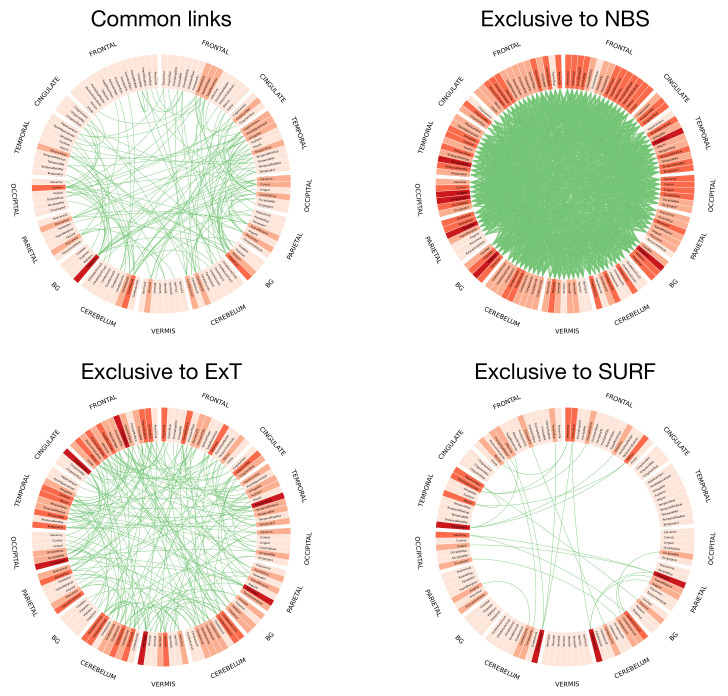
Graphical representation of the links common and exclusive to the three best performing methods, in terms of AUC. Specifically, the top left panel reports the circular representation of the links common to NBS, ExT and SURF; while the other three, the set of links that have exclusively been selected by respectively NBS, ExT and SURF. Nodes (i.e., of Regions of Interest, see Section 3.1) are labelled according to the corresponding name in the AAL parcellation; colors indicate their degree, from light (no or few connections) to dark shades (maximum degree). The four panels have been prepared using the Circos software [91].

Figure 6 further depicts the inter-stability, i.e., how overlapping are the sets of retained links yielded by pairs of methods, again measured with the metric *D*. In a way similar to Figure 5, it can be appreciated that values are generally low; for instance, two of the best performing methods (NBS and AnovaNet) have a D≈0.54, i.e., they agree on approximately 50% of the links. Such heterogeneity is further depicted in Figure 7, reporting the circular plot of the common links among the three best performing methods (top left panel), and the links exclusive to these three methods. It can be appreciated that common links are distributed among all brain areas, with the only exception being the thalamus, recognised as important by the three methods. This latter result is well aligned with the recognised importance of thalamic circuitry in Schizophrenia [88,89,90]. On the other hand, the number of links exclusive to each method vary between few tens (for SURF) to several hundreds (NBS). The implications of the heterogeneity in the sets of selected links will further be discussed in the conclusions.

### 4.3. Computational Cost

**Figure 8:**
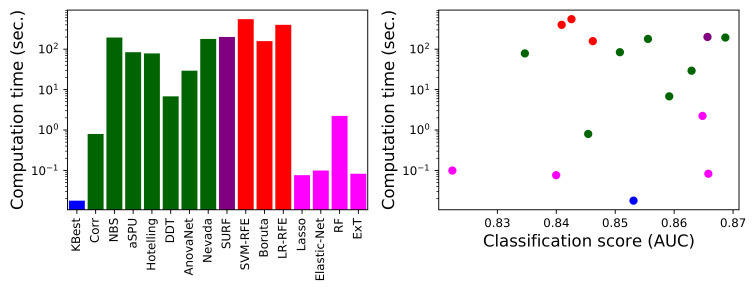
Computational cost analysis. (**Left**) Computation time for each link selection method (excluding the three underperforming ones), measured in seconds. (**Right**) Scatter plot of the computation time as a function of the performance (in terms of AUC) of each method. Colour code as per Figure 1.

As a final issue, of major importance in a real-world environment where the objective may be to analyse large-scale data sets, we here compare the computational cost of the 16 best performing methods. Such cost has been estimated as the time required to extract the best set of links, excluding the time required for the subsequent classification and validation, using a four-cores Intel^®^ i7-8565U CPU at 1.8 GHz, 8 GB RAM machine. Results are presented in Figure 8, both independently (left panel) and as a function of the classification score obtained with each method (right panel).

The large differences between methods stand out, spanning almost five orders of magnitude-note that results are depicted using a logarithmic scale. Some methods, like KBest, require a fraction of a second to yield their results, while other, e.g., Nevada, tens of minutes. It is interesting to note that statistical methods are usually not the fastest ones; even if they may be conceptually simpler, they usually require the creation of random ensembles to obtain *p*-values, resulting in a substantial computational cost. On the other hand, the opposite is true for the embedded machine learning methods. This can be due to two complementary reasons: firstly, that they do not require extra computational effort beyond the training of the model itself; and secondly, that their implementation, included in standard and well curated libraries like the scikit-learn package in Python, is especially tuned for efficiency.

### 4.4. Generalisability of Results

One final important aspect to be considered is how general the results here reported are; in other words, can a practitioner be confident that, for instance, the best link selection model detected in Figure 2 will also be the best performing one when applied to a different dataset? Note that the generalisability of neuroscience results, and especially of those obtained through network representations, is an ongoing field of research; and that a lack of generalisability is usually recognised, mainly due to the many arbitrary steps involved in data preprocessing that may affect results in a unpredictable way [92,93,94].

**Figure 9:**
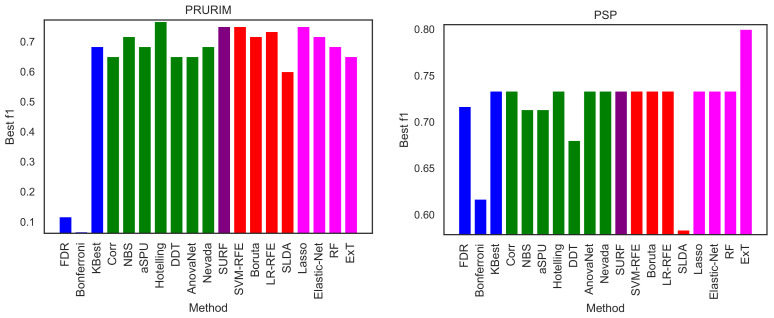
Best classification scores, in terms of the F1 metric, for the PRURIM (**left panel**) and PSP (**right panel**) datasets.

**Figure 10:**
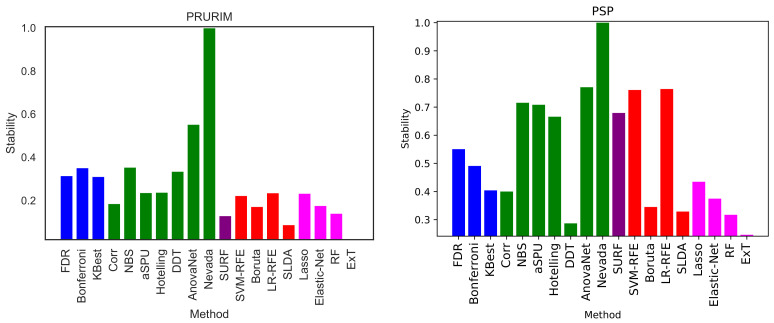
Intra-stability *D* of each method, for the PRURIM (**left panel**) and PSP (**right panel**) datasets. See Equation (3) for a definition of the metric *D*.

We here perform a basic test by executing the same exercise over the two remaining brain networks datasets, i.e., PRURIM (see Section 3.1.2) and PSP (Section 3.1.3). Specifically, Figure 9 reports the classification score, in terms of F1, for the two datasets; and Figure 10 the corresponding intra-method stability. Results are generally compatible with those reported in Figure 2 and Figure 5. Most methods achieve comparable classification scores, while FDR, Bonferroni and SLDA are underperforming. It can also be appreciated that results are more flat in the case of the PSP dataset, i.e., the F1 presents very similar values across all methods; this is due to the limited size of this dataset, which only comprises 64 networks of 27 nodes.

## 5. Discussion and Conclusions

When analysing networks representing brain dynamics in different conditions or pathologies, the use of some kind of selection method is an appealing concept. Using the whole network can be computationally and conceptually challenging, especially in the case of fMRI, which in its raw form may include $>10^{4}$ nodes and $>10^{8}$ links. Processing those large networks does not only require substantial computational resources; but it also makes it difficult to pinpoint what are the relevant elements characterising a pathology or condition. In a way similar to feature selection in machine learning, selecting and retaining only a subset of those nodes and links yields an easier to interpret picture, while not discarding any relevant information. It further helps increasing the statistical significance of results, and may improve subsequent processing, as e.g., classification tasks. In this contribution we have compared a large set of different link selection methods, drawn from statistics and machine learning, and compared their performance in terms of performance (i.e., how much information is retained at the end, measured through a classification task), stability and computational cost. The ranking of the top-5 methods according to each one of these criteria is reported in Table 1.

The most important and somewhat surprising result is that, except for a few methods (i.e., SLDA, FDR and Bonferroni), all others yields a similar classification score; in other words, and in spite of their internal differences and underlying hypotheses, the information they retain is almost the same—see Figure 2 and the first column of Table 1. Within this group, the difference between the best (NBS) and worst (Elastic-Net) performing ones is actually of the same magnitude of the standard deviation of scores obtained in different iterations of their cross-validation; the difference is thus similar to the one expected when processing different data sets, and thus not significant. Regarding the ranking of methods, the best scoring method (i.e., NBS) was also found to be the best one in previous, smaller scale comparisons, as for instance in [22]. As a second point, it is worth noting that the classification score obtained using the filtered sets of links is equal, and in some cases even larger, than the one corresponding to the use of the full data set. Therefore, deleting uninformative links does not only simplify the problem, but even increase our capacity of extracting conclusions from it. While this is a well-known idea in machine learning, its application in neuroscience has so far been limited.

Moving to the intra-stability of each selection method, i.e., its capacity of consistently yielding the same set of links, results are generally low—see Figure 5 and the second column of Table 1. Nevada stands out as an exception; yet, this is due to the large number of links it retains, and it is thus not significant. We hypothesise that this may due to two different reasons: either the methods are not stable, i.e., they are designed in a way that yields unstable results when applied to brain networks; or the network themselves are unstable. This second possibility implies that the variability between people is large, such that a condition may manifest in different parts of the brain networks in different people. While this is not at odd, but actually aligns well with previous research works on brain network generalisability [95,96,97], it is worth noting that the data and analyses here presented cannot draw a conclusive answer to this problem.

The stability across link selection methods, i.e., the inter-stability, is also low - see Figure 6. In other words, while different methods may retain all relevant information for the task at hand, such information is not encoded by the same links. This suggests an interesting picture, in which relevant information is encoded in a redundant way in different parts of the brain network, parts that are selected by the methods according to different criteria. In other words, a pathology may affect two sets of links *s* and $s^{\prime }$, with little overlap between them; choosing any one of them is thus equivalent, and no added value is obtained by considering the two sets together. While this resembles the concept of brain’s default mode networks [98,99], to the best of our knowledge the presence of pathology-related complementary sub-networks has not previously been discussed in the literature.

How do these results contribute to answering the main question tackled in this contribution, i.e., what is the best method for link selection in brain networks? In general terms, the conclusion is that, excluding a few exceptions, any method is good, and indeed a selection step is even necessary to improve our understanding of the brain. Factoring in the results regarding stability and computational costs, two cases can be defined. When the priority is to obtain the best classification score, NBS is the best solution; it yields scores marginally better than its peers and a good stability, albeit at the cost of a high computational complexity. On the other hand, in case of large data sets or problems in which the time required to obtain an answer is a concern, one may resort to ExT or KBest: their computational cost is three orders of magnitude smaller and they yield smaller output sets, the price being a slightly lower precision in the subsequent classification and a much reduced stability in the selected links.

As a final note, it is important to discuss some limitations of the present study. First of all, it is based on the data of three datasets, including information about three pathologies (and matched control subjects). The generalisability of the conclusions here presented to other datasets and pathologies is not guaranteed, even though pertinent actions have been taken towards it—e.g., use of cross-validations and resampling of subjects, and use of different sample sizes and thresholdings. On the other hand, COBRE is a well-known data set, used as reference in many neuroscience studies, e.g., [100,101,102,103]; the presented results are thus in any case of relevance to the neuroscience community. Secondly, the reconstruction of functional brain networks is a process entailing many steps, in which several decisions (e.g., use of different parameters or methods) have to be taken, and usually prone to a large degree of subjectiveness [104]. This results in different conclusions drawn from a same data set [92,93,94]; and, consequently, the possibility that the results here presented are dependent on the choices made in the network reconstruction process. The researcher performing any analysis on neuroimaging data should therefore always practice caution, also in the choice of the selection method, for instance comparing a couple of them. Thirdly, there is a high heterogeneity in the codes used to evaluate each method: some of them are included in recognised and well-maintained libraries, others have been released by the authors of the methods, and some have been developed specifically for this contribution. As such, the comparison of the computational costs (Figure 8) must be taken with due caution, as some methods may be susceptible to substantial optimisations. Still, results here presented are representative of what a researcher with average coding skills may expect to face; and further suggest additional development tasks, e.g., the creation of a unified software library of link selection methods.

To conclude, this contribution focused on two wide families of link selection methods, i.e., statistical and machine learning ones. The interested reader must be aware that other alternatives can also be found. To illustrate, some methods based on Deep Learning have been proposed, see for instance [105,106,107]; while DL approaches generally yield excellent results, they are also black-boxes, i.e., they do not facilitate inspecting or making explicit their internal logic. The comparison of these DL methods with statistics and machine learning ones, both in terms of performance and computational cost, shall be the focus of future research works. Secondly, machine learning is a mature yet active field of research, with many fruitful applications to neuroscience problems [108,109]; not surprisingly many studies have been published, both targeting specific pathologies [110,111,112,113,114], and focusing on alternative methods for link selection [115,116,117]. Finally, the reader should be aware of the large body of literature related to the analysis of brain networks [5,6]; while most of the time the whole network is considered and is analysed using macro-scale topological metrics, some studies have also identified specific subnetworks or subsets of links using network science principles [112,118,119,120,121,122,123].

## Figures and Tables

**Table 1:** Ranking of the top-5 methods, according to their AUC, stability *D* and computational cost, as obtained for the COBRE data set.

Best AUC		Best Stability (*D*)		Best Computational Cost (sec.)	
NBS	(0.869)	Nevada	(1.000)	KBest	(0.018)
ExT	(0.866)	FDR	(0.711)	Lasso	(0.076)
SURF	(0.866)	Bonferroni	(0.602)	ExT	(0.083)
RF	(0.865)	AnovaNet	(0.536)	Elastic-Net	(0.099)
DDT	(0.859)	NBS	(0.426)	Corr	(0.798)

## Data Availability

The COBRE dataset is available at http://fcon_1000.projects.nitrc.org/indi/retro/cobre.html (accessed on 15 February 2021) and processed brain networks presented here are available upon request of the authors. The PRURIM and PSP datasets are available at http://umcd.humanconnectomeproject.org/umcd/default/browse_studies (accessed on 15 February 2021).
